# Vulcanizing Rubber

**Published:** 1894-12

**Authors:** Geo. B. Snow


### Vulcanizing Rubber.

BY DR. GEO. B. SNOW.

Re ıd before the 38th Annual Session of the Michigan Dental Association.

Dr. Geo. B. Snow, of Buffalo, N. Y., presented some samples of vulcanized rubber, and showed what the physical changes are that take place in that material during the process of vulcanization. The samples, both by their change in shape and by their increased specific gravity, showed that their mass was perceptibly smaller after vulcanization, the change differing according to their composition; those composed of pure rubber and sulphur shrinking more than those containing a large percentage of foreign matter; the scale running from pure black to pink, the latter showing the least change. The difference in the specific gravity of the samples showed the change by vulcanization to run from about 6 per cent. for pure black rubber to about 3 per cent. for pink. Attention was drawn to the fact that the heretofore unaccountable mishaps which occur in vulcanizing, such as loose teeth, vacant spaces under the shoulders of bicuspids and molars, and under section teeth, are easily explained when the shrinkage of the rubber is taken into account; their occurrence being much more frequent in the use of black or pure rubber than the colored ones.

The subject of the expansion of rubber by heat was then taken up, and specimens were exhibited which showed that its expansion from 212 to 320 degrees is fully equal to its shrinkage in vulcanizing; the deduction following that if the flask was properly packed, and closed in hot water, it would contain rubber enough to make a sound plate if none of it were allowed to escape. As gate-ways are usually cut, a constant escape of rubber is going on while the vulcanizer is reaching the vulcanizing point, with the result that if there was no change in it by vulcanizing there must still be a deficiency in the mold when it cooled to the amount of the escape into the gate-ways. A strong plea was then made for the use of spring pressure upon the flask while vulcanizing, and it was shown that by cutting a circular overflow chamber around the mold, leaving a narrow edge of plaster around it perfect, so that the rubber would be imprisoned when the flask was closed, and by using spring pressure upon the flask in the vulcanizer, the necessary conditions would be fulfilled, and the results would be very much more satisfactory, both to the operator and the patient.

Two plates were shown, which were vulcanized in the College Laboratory at Ann Arbor, one in the ordinary way, and the other according to the process described. The two plates were counterparts, being made upon duplicate models, with section teeth set at a considerable distance from the alveolar ridge and mounted in black rubber. When vulcanized, a bicuspid block was broken from each in order to show the difference between them. The one vulcanized in the ordinary way had a space under the teeth, so that a Swiss saw could be passed under all of them, even to the molars on the opposite side and the pins of the broken bicuspid block were perceptibly loose in the rubber. The other plate showed the rubber to be in perfect contact with the teeth and the pins to be held firmly.

The changes in the plate after vulcanizing, by the contraction of the rubber in cooling were then noticed, and it was shown that they were much more marked when section teeth were used, on account of the very slight contractile power of the porcelain. The sections form an arch which diminishes in width by the contraction of the rubber inside it, with the effect of causing the plate to rock upon the hard palate. The plate is, in fact, a little too narrow for the mouth; and if it becomes necessary to re-vulcanize it, the difference is often perceptible, and lower plates, after successive repairs and re-vulcanizations, will become so loose as to be unwearable. It was shown that the difficulty could be overcome by heating the plate enough to soften the rubber while its heels were forced apart by a stay, thus widening the arch and restoring it to its original dimensions.

